# The hard problem of consciousness—A perspective from holistic philosophy

**DOI:** 10.3389/fnins.2022.975281

**Published:** 2022-10-25

**Authors:** Jicheng Chen, Linlin Chen

**Affiliations:** Department of Vasculocardiology, Shenzhen Longhua District Central Hospital, Guangdong Medical University, Shenzhen, China

**Keywords:** hard problem of consciousness, reductionism, holistic philosophy, perception, contradiction, free energy principle, quantum mechanics

## Abstract

Based on a material view and reductionism, science has achieved great success. These cognitive paradigms treat the external as an objective existence and ignore internal consciousness. However, this cognitive paradigm, which we take for granted, has also led to some dilemmas related to consciousness in biology and physics. Together, these phenomena reveal the interaction and inseparable side of matter and consciousness (or body and mind) rather than the absolute opposition. However, a material view that describes matter and consciousness in opposition cannot explain the underlying principle, which causes a gap in interpretation. For example, consciousness is believed to be the key to influencing wave function collapse (reality), but there is a lack of a scientific model to study how this happens. In this study, we reveal that the theory of scientific cognition exhibits a paradigm shift in terms of perception. This tendency implies that reconciling the relationship between matter and consciousness requires an abstract theoretical model that is not based on physical forms. We propose that the holistic cognitive paradigm offers a potential solution to reconcile the dilemmas and can be scientifically proven. In contrast to the material view, the holistic cognitive paradigm is based on the objective contradictory nature of perception rather than the external physical characteristics. This cognitive paradigm relies on perception and experience (not observation) and summarizes all existence into two abstract contradictory perceptual states (Yin-Yang). Matter and consciousness can be seen as two different states of perception, unified in perception rather than in opposition. This abstract perspective offers a distinction from the material view, which is also the key to falsification, and the occurrence of an event is inseparable from the irrational state of the observer’s conscious perception. Alternatively, from the material view, the event is random and has nothing to do with perception. We hope that this study can provide some new enlightenment for the scientific coordination of the opposing relationship between matter and consciousness.

## Introduction

In the past few 100 years, biology and physics have achieved remarkable success. On the basis of material view and reductionism, we regarded the external as an objective being and ignored the inner conscious experience. The natural phenomena and laws are described by observation and statistics and the macroscopic phenomena are explained by microscopic quantum. For example, the phenomenon of life is explained by cells and the origin of the universe is explained by microscopic quantum. We have long been accustomed to deploying the cognitive paradigm of reductionism. Its underlying assumptions and methods are taken for granted. However, this cognitive paradigm has brought about a series of puzzles about consciousness in both biology and physics, reflecting its limitations.

Over the past few decades, neural and cognitive scientists have made remarkable progress in studying consciousness from a physical level (Dehaene and Changeux, 2011; Boly et al., 2013; Owen, 2019). Koch et al. (2016) argue that we are now at a point where we can understand consciousness in a scientific way, such as neuronal correlates of consciousness (NCC), and not as a philosophical question, especially in the field of visual consciousness (Crick and Koch, 1998; Koch et al., 2016), and these represent the functional side of consciousness research. However, subjective experiences cannot be explained from an objective standpoint. Relatedly, how do organisms produce the meaning of life that we experience, and how it relates to the brain (the mind–body problem) (Reddy, 2016; Levin, 2020)? This represents the “hard problem of consciousness” (Chalmers, 1998; Solms, 2014, 2021; Solms and Friston, 2018).

From another point of view, similar to the above problem, there is a contradiction between free will and causality based on time and space, which cannot be currently explained by reductionism (Heisenberg, 2009; Rappaport, 2011; Hillman, 2018). For humans, if our brain produces certain thoughts, we can detect the electrical activity in the corresponding regions of the brain with instruments, but we do not have an idea what causes nerve cells to become excited. We do not get excited by an external electrode stimulation, which is perceptually called free will.

We establish causality based on time and space but, in an experiment like this, the electrical excitation of the brain’s nerves is triggered by invisible thoughts or motivation that we think of as autonomous without any physical cause. But we do not know exactly how invisible thoughts lead to physical changes in the brain. This feature of consciousness undoubtedly challenges the idea of causality, dependent on space and time. Is the sense of freedom we perceive not subject to the laws of the physical world? If we attribute the neuroelectric excitation to the external physical environment, it means that we are like a robot, free will is just a mechanical reflection of the environment, a kind of illusion. Although there is some neurobiological evidence against the nature of free will, the evidence is not convincing. More importantly, if free will is an illusion, how do we explain the meaning of life? (Brass et al., 2019; Lavazza, 2019).

The cognitive paradigm of material view and reductionism also leads to the puzzle of consciousness in quantum physics. Matter and consciousness, which used to be philosophical issues, have become concrete scientific problems (Frank, 2015). Quantum mechanics has revealed some puzzling microscopic phenomena, such as wave-particle duality and quantum entanglement. These phenomena have challenged classical thinking regarding the objective physical reality and suggest an inseparable aspect of matter and consciousness, in which we cannot treat consciousness as an illusion. To solve the core problem of how quantum random collapse produces a well-ordered world, scientists have focused on consciousness as the key. John von Neumann argued that only consciousness could eventually collapse the wave function to produce a definite reality (Neumann, 2020). Eugene Paul Wigner argued that the role of conscious creatures in quantum mechanics must be different from that of inanimate measuring devices (Wigner et al., 1992). In 2007, Robert Lanza and Bob Berman came up with a new concept termed biocentrism (Lanza, 2012). They proposed that order or reality requires the presence of a conscious observer. However, how consciousness causes wave function collapse (or affects reality) remains unclear.

In conclusion, we think that although these consciousness-related puzzles take different forms in different disciplines, what they have in common is that they jointly reveal that matter and consciousness (body-mind) interact and cannot be separated, but they lack a scientific explanation of the underlying principle and mechanism. For example, how can abstract subjective experiences lead to physical neural excitation (we cannot observe any medium)? How does consciousness affect wave function collapse? The cognitive paradigm of the material view, which puts matter and consciousness in opposition, will lead to such gaps in interpretation. We propose there is another cognitive paradigm that can reconcile the antagonistic relationship between matter and consciousness and reconcile these dilemmas.

Currently, scientists are trying to build models to understand the nature of consciousness (Seth and Bayne, 2022). The free energy principle proposed by Friston and Stephan (2007) is applied to explain this puzzle and it has become a compelling solution (Solms, 2018, 2021). The precursor to the free-energy principle was a way of describing how the brain works. At every level, the brain’s prediction of what the most likely experience will be in a given environment is compared with the actual information received from the senses. If the prediction is not correct, then higher levels of the nervous system are required (Friston and Stephan, 2007; Ramstead et al., 2018). The free energy principle describes the mind–brain system as any other adaptive biological system, connecting psychological sciences, neuroscience, and related fields in confluence and synergy with psychoanalytic concepts (Cieri and Esposito, 2019). In addition, there are some other well-known theories. Integrated information theory (IIT), developed by Tononi and collaborators, focuses on the objectivity of subjective experience itself (Koch et al., 2016). The orchestrated objective reduction (or “Orch OR”) model, developed by Hameroff and Penrose, has suggested that consciousness is the result of the collapse of wave functions caused by quantum gravity in microtubules (Hameroff, 2012; Hameroff and Penrose, 2014). These hypotheses offer a deeper insight into the understanding of the phenomenal aspect of consciousness (Rees et al., 2002).

Philosophical perspective may offer inspiration for scientific studies and provide theoretical foundations for understanding the relationship between matter and consciousness (or the nature of consciousness; Churchland and Churchland, 1997; Sturm, 2012). The confusion afflicting physics today has led scientists to understand the universe from a more holistic perspective. Niels Bohr believed that the Taiji diagram (the logo of holistic philosophy) contained the principle of wave-particle duality (Capra, 2000), and quantum physicist Bohm (2004) tried to explain the origins of order from the perspective of wholeness in his ontological picture of the universe. However, we still need to build a more detailed theoretical model of consciousness that can be described scientifically on the basis of a deeper understanding of holistic philosophy.

There are significant cultural and cognitive differences between the East and West (Wang et al., 2021). The material view is not the only cognitive paradigm in which we describe the movement and development of the universe, *the Book of Changes* and *Tao Te Ching* tend to understand the world from a holistic perspective (Yutang, 1948).

This holistic philosophy has profoundly affected different cultural forms of the East and provided a series of effective social applications (Liu, 2008; Kafatos and Yang, 2016). As an important work from the perspective of holistic philosophy, this study discusses our understanding of *Tao Te Ching*. We propose that the theory of scientific cognition exhibits a paradigm shift in terms of perception. With a tendency implying that reconciling the dilemma of consciousness requires an abstract theoretical model that is not based on physical forms, the Taiji diagram in the philosophy of the holistic view is a candidate. We propose that the holistic perspective provides a potential solution and new inspirations to solve current reductionism-based scientific dilemmas.

Objectivity is the foundation for establishing a theoretical system in both the material view and holistic view. The cognitive paradigm of holistic philosophy is based on the basic objectivity of perception, which shows the objective nature of contradiction beyond the control of the individual, but intuitively, we think of it as subjective or as belonging to an individual. Although objectivity is abstract, it is the basis and key to establishing a holistic description system, just like our description of the objectivity of different physical quantities. The holistic view relies on conscious experience (rather than observation) and reduces everything to two abstract perceptual states: Yin-Yang. We regard matter and consciousness as two contradictory perceptual states that are unified in perception. Their unity implies that the inner and the outer are not absolute opposites, but that there is an interconvert relationship between the two. This perspective avoids the dilemma of consciousness caused by the emphasis of the material view on the external objective description. We will elaborate on the holistic philosophy in the following paragraphs.

## Holistic philosophy

### Different explanations of the origin and evolution of the universe from *Tao Te Ching*

*Tao Te Ching*, written by Lao Zi, has had a profound influence worldwide. It offers a representative interpretation of holistic philosophy, although there is no unified interpretation of the book. In this part, we first introduce the core ideas of the holistic philosophy in the *Tao Te Ching*. We will discuss this in detail with some examples in the following chapters. Its first and most important chapter includes a brief exposition of the origin of *everything*:

The Tao that can be told of, Is not the Absolute Tao;/The Names that can be given, Are not Absolute Names./The Nameless is the origin of Heaven and Earth, The Named is the Mother of All Things./Therefore: Oftentimes, one strips oneself of passion/In order to see the Secret of Life; Oftentimes, one regards life with passion,/In order to see its manifest forms./These two (the Secret and its manifestations) Are (in their nature) the same;/They are given different names, When they become manifest./They may both be called the Cosmic Mystery:/Reaching from the Mystery into the Deeper Mystery, Is the Gate to the Secret of all life (Yutang, 1948).

We think this chapter has the following three meanings:

### Existence is relative and an objective material entity independent of perception is essentially indescribable

The creation of everything (reality or a phenomenon to be described, such as the state of a particle rather than a particle entity) and perception occur simultaneously and irreplaceably; they are two sides of the same coin. This differs greatly from the objective observations that we assume in intuition and basic scientific assumptions. According to our understanding, a state of being always requires symmetric physical descriptors, such as up and down, large and small, light and heavy, and more and less. An alphabet without letters cannot be described, and, in the same way, being or matter cannot exist without these contradictory descriptors (difference), which cannot exist independently from perception. In other words, an objective material entity or physical descriptor (state) cannot exist independent of perception (Lanza, 2012). For example, the statement “our bodies are made up of cells,” is usually thought of as an objective phenomenon, and cells are an objective existence that is the same for everyone, whether you know or observe them or not. However, according to our understanding of *Tao Te Ching*, the observed cells (“The Named” in *Tao Te Ching*) and the feeling of the person describing the cells (“The Nameless” in *Tao Te Ching*, cannot be concretized) are two objective states of existence that appear simultaneously and cannot be replaced by one another.

**FIGURE 1:**
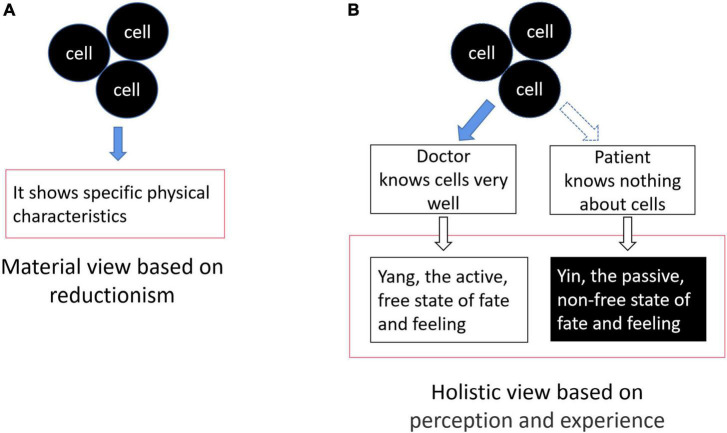
The material view and the holistic view put different emphasis on objectivity. **(A)** Cells are considered objective material entities that are the same for everyone whether you know/observe it or not. This paradigm focuses on external objectivity based on observation. **(B)** Understanding the rules of the cell can cure disease, although this effect is universally applicable, however, the different experience on the “same and objective” cells results in different fates. Different “fates” are also another aspect of objectivity. This paradigm emphasizes the objectivity of feelings and experiences rather than external physical forms.

On one hand, cells exhibit external physical characteristics, such as mass and size, which can be observed, and the objectivity of cells is thus recognized. On the other, a doctor who knows a lot about cells and a patient who is being treated have different perceptions of the “cell” (a material entity regarded as objective, which forms different perceptual states for doctor and patient) that result in different realities or state of feelings caused by their different roles (the doctor and the patient are, respectively, active or passive reality). This has important meaning beyond physical form; although it cannot be described concretely, it is also an objective aspect. Therefore, if the cell is defined as a material entity that is objective or the same to everyone, this sort of cognition is one-sided. Although we can explain the body in terms of the laws of cells based on reductionism, we also know that a group of cells does not equal a person. Subjective experience cannot be explained (Figure 1). A holistic view focuses on the objectivity of these abstract perceived states (the meaning of feeling and subjective experience) rather than external physical forms. It can be used to build a different descriptive system depending on the perception that completely differs from material views. Many social applications of the East, such as traditional Chinese medicine, acupuncture, architectural design styles, and culture overall, derive from this philosophical view.

### An objective law describing everything independent of perception does not exist too

While we may ponder whether the world is deterministic or non-deterministic, which remains unclear, holistic philosophy may provide a reference point for this question. When we undertake scientific explorations, we see ourselves as observers based on the distinction between our physical form and external objects, which we take for granted. According to reductionism, we have succeeded in explaining macroscopic phenomena in terms of microscopic quanta. This leads us to believe that we can construct a theory explaining *everything* based on reductionism. Following this assumption, external objective laws and the movement of the universe have nothing to do with perception. However, according to our understanding of holistic philosophy, a theory of *everything* cannot exist independently of perception. An objective law describing the evolution of everything (reality) independent of perception does not exist, and we cannot make objective remarks as independent observers or separate perception from the laws of nature. Conscious experiences and preferences in feelings participate in the creation of order/reality. We will discuss this assumption in detail in the next chapter.

### The way order and reality occur depends on the state of perception

In Chapter 42 of *Tao Te Ching*, Lao Zi defined the development trend of *everything* as follows:

“Out of Tao, One is born;/Out of One, Two;/Out of Two, Three;/Out of Three, the created universe./The created universe carries the yin at its back, and the yang in front;/Through the union of the pervading principles it reaches harmony” (Yutang, 1948).

As per our understanding, based on the perceived differences in the positions of the two sides of a conflict, the development of reality always tends toward the “good” side of feelings (it is a relative concept that depends on perception), such as reasonableness, balance, equality, unity, and fairness (the created universe carries the Yin at its back and the Yang in front). Otherwise, it will increasingly encounter resistance, making this form untenable and leading to either a collapse or a shift to the opposition.

In general, the movement of everything is always from opposition to unity. The frame of reference that influences reality is an internal rather than an external concrete frame of reference. In other words, causality is not external but inseparable from perception, being a relative concept depending on the state of perception.

If body and mind are two appearances (aspects) of the same underlying thing, then what stuff is the underlying thing made of? In other words, using the analogy of thunder and lightning, what is the metapsychological equivalent of “electricity” i.e., the thing that gives rise to thunder and lightning, both? (Solms, 2018).

According to holistic philosophy, matter and consciousness (body–mind, external or internal) are not opposites, but two sides of the same coin. They are seen as two states that can be separated by perception (represented by Yin-Yang), which can be used to describe the evolution of reality in a paradigm shift way. Rather than relying on observations and statistics, this kind of description relies on conscious experience. In contrast to the material view, the unity of matter and consciousness at the level of perception also implies that the occurrence of reality is an inseparable process from conscious experience.

For example, over the process of evolution, the nests that ants build to adapt to their environment must have changed dramatically, and we are using the ant nest as a metaphor for reality (the result of an event or phenomenon being observed). If we observe a nest of ants and replace any individual ant, we will find that the construction of the ant nest will not be affected. We think of the individual as unimportant; ants build nests by instinct, which is unconscious behavior. This is just a description of a static phenomenon, the individual conscious experience of ants is ignored. However, we know (through perception and empathy, rather than observation) that the structure of ant nests also evolves and this process is not random; it depends on the constant adjustment of the invisible individual’s perceptual state to adapt to the external environment.

In other words, reality comes from the interaction of the individual (inner feelings and experiences) with the external environment and depends on the preferences or tendencies in perception. The irrational sense (to seek a more harmonious state of feeling) is a factor in the creation of observed reality, although this is not visible to the observer. It is an abstract and relative concept. This is an important difference between holism and material view. As an observer, reductionism tries to find causes from the outside and descriptions of concrete objects’ motion. Following a holistic view, perception is an objective being, the occurrence of reality is inseparable from the state of conscious experience (inner). Although we use ants as an example, according to holism, this description applies to *everything*, even if the object is a microparticle or abiotic (the created universe carries the “Yin” at its back and the “Yang” in front).

**FIGURE 2:**
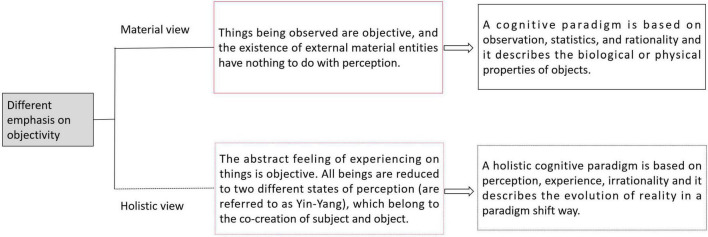
Different characteristics of material view and holistic view. In these two different cognitive paradigms, we propose that the cognitive paradigm based on the view of matter has significant, concrete and widely disseminated characteristics. Holism, on the other hand, is inaccurate and abstract because of its reliance on experience and perception. The social applications derived from it have similar characteristics, focusing on the internal influence of external reality and purpose rather than tools invention. For example, traditional Chinese medicine, tenon and mortise, acupuncture and moxibustion, architectural style and soon. This paper tries to put forward a hypothesis that can be scientifically tested to prove the rationality of this philosophical paradigm and provide reference to solve the paradox of consciousness in biology and physics.

This is not to say that inanimate objects or particles have perception or consciousness, but that the state of perception itself is objective. It is an objective existence born together from subject and object. Unlike material views, which treat external material entities as objective beings, in a holistic view, the state of perception (Yin-Yang; is the individual’s reality passive or active, free or not free, purpose achieved or not achieved) and the preference of feeling is the most objective existence. It does not depend on external physical forms to describe objects, but different realities (reality is passive/Yin or active/Yang) experienced through perception. Therefore, the object described through a holistic view is abstract reality (reality is relative, and the “same” phenomenon means different things to different people) inseparable from perception, strongly differing from material views (Figure 2). Why can reality be described? In later chapters, we will discuss the objectivity of perception, its character beyond individual control determines the basis on which reality can be described.

## Discussion on the development of characteristics of scientific cognition

### Scientific cognition shows a significant paradigm shift trend from opposition to unity in terms of perception

In (section “The paradigm-shifting trends of reality depend on irrational feelings”) we propose that an objective law describing everything independent of perception does not exist. Here, we will discuss in detail why the process of scientific exploration is not just a process of objective observation.

**FIGURE 3:**
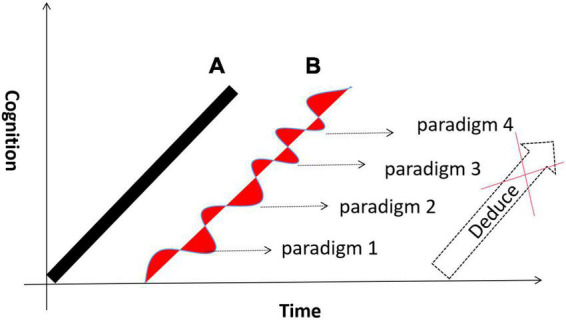
The development characteristics of scientific cognition. **(A)** Theoretically, the development of scientific cognition should be a linear process. **(B)** Thomas Kuhn thought that the development of scientific cognition is a non-linear paradigm shift process, and there is not a logical relation between the new paradigm and the old paradigm.

The term “paradigm shift” was first coined by the twentieth-century philosopher Thomas Kuhn. In Kuhn’s view, a paradigm is a specific knowledge system formed by a series of results obtained from scientific research (Kuhn, 2004). The widespread acceptance of a paradigm indicates the maturation of a scientific field. When existing paradigms fail to explain certain natural phenomena, new paradigms that can explain them emerge. Kuhn also believed that there is nological relation between the new paradigm and the old paradigm. In other words, new theoretical paradigms cannot be deduced from old paradigms by relying on logic (Figure 3). However, if we analyze key scientific theories of physics and biology from the perspective of their historical development, we will find that the cognitive pattern (we are concerned not with the mathematical or physical form of the theories but with their abstract meaning in perception) abstracted by these revealed phenomena show surprising similarities and development trends.

#### Physics

Before Copernicus, the Earth was thought to be the center of the universe. In 1543, Copernicus formally proposed the heliocentric theory, placing the sun at the center of the universe. In terms of spatial arrangement, this theory overturned the self-centered (human) cognitive model.

In 1687, Newton proposed the law of universal gravitation, which became the cornerstone of classical physics. This allows us to accurately describe the motion of objects based on the principle of force interactions. A simple linear causal cognitive model is created based on the opposition between time and space. Newton’s view of space and time dominated physics for over 200 years until Einstein’s theory of relativity deepened our understanding of Time and Space (quality and energy), which do not exist independently and are naturally linked. This discovery overturned the opposing relationship of time and space and indicated the universality of connections on a material level. Nonetheless, the observer and the object remained in a state of opposition (more basic forms of opposition).

In the nineteenth century, scientists came together to develop quantum theory, represented by the phenomenon of wave-particle duality and quantum entanglement. At the microscopic quantum level, the description of a quantum state requires a conscious observer, and the observer and the object (subject and object) are inseparable. It further deepened the scope of a universal connection based on the indivisibility of relativistic space-time, thereby challenging the most basic scientific assumptions about the distinction between subject and object based on physical form opposites.

#### Biology

In biology, scientific cognition developed similarly. In the seventeenth century, species were believed to be created by God and human beings had a core status in nature. However, in 1858, Charles Darwin and Alfred Russel Wallace proposed the theory of natural selection at the Linnean Society in London, explaining the orderly evolution of biological species, including humans. This theory subverted the self/human-centered cognitive paradigm for positioning biological species in nature.

Before the work of the Austrian biologist Gregor Johann Mendel, people’s understanding of biological traits was vague and abstract. Biological traits showed the dual characteristics of heredity and variation, similar to the cognition of the motion of stars and objects before Newton’s theory of universal gravitation. In the middle of the nineteenth century, findings in molecular biology revealed that interactions between ligands and receptors produce information transfers that form the basis of microscopic activities. In 1865, Mendel revealed the laws of segregation and independent assortment in genetics (dominant and recessive genes) following 8 years of experiments with hybrid peas. Based on the principle of interaction, biology has moved away from the abstract perception of phenomena to a concrete description, to a more unified understanding of biological traits at the microlevel.

Microbiological studies in recent decades have revealed a very complex network of molecular interactions. Although the importance of molecules varies, in essence, there is no simple linear cause and effect in the determination of biological phenotypes, and compensatory effects are common among molecules. At the microlevel, the transition has moved from simple linear causal cognition to non-linear universal molecular interactions.

In 1992, the discovery of mirror neurons further demonstrated that we learn about the world not just by independent observation but through perception and imitation (Dapretto et al., 2006; Falck-Ytter et al., 2006; Rizzolatti and Sinigaglia, 2016). For example, monkeys watching one another eat have neural activity in the same regions of the brain. This reflects the non-absoluteness and indivisibility of the role of the observer and the observed object, which is a relationship of inclusion, analogous to quantum phenomena in physics from an abstract meaning. By describing the developmental process from the core scientific theories mentioned above, we can roughly conclude that scientific cognition has the following characteristics.

First, from self/human-centered cognition to self/humans are not special cognitive conclusions/trends and from the antagonistic relationship based on physical forms to the indivisibility of the subject and object (internal and external, consciousness and matter). We are aware of our existence because of the differences in external physical characteristics with the outside world, and we define ourselves/human beings in terms of these physical or biological characteristics. In the development of science, our cognition of these descriptive quantities is also a synchronous process of redefining ourselves/human beings. This process shows a trend from the perception that the self/human is special to the perception that the self is not special (or from self-centeredness to the cognitive conclusion of the self/human is not special). For example, we originally defined ourselves in terms of unique biological traits, distinguishing ourselves from other living or non-living beings through biological trait differences. Then, the theory of natural selection (the form of contradiction is the relationship between the individual and environment) broke down this notion of the special status of humans in nature. Subsequent scientific exploration revealed that these traits are non-special and can be explained uniformly at the microlevel by genes and proteins (proteins are extrinsic exhibitors and genes are arbiters).

**FIGURE 4:**
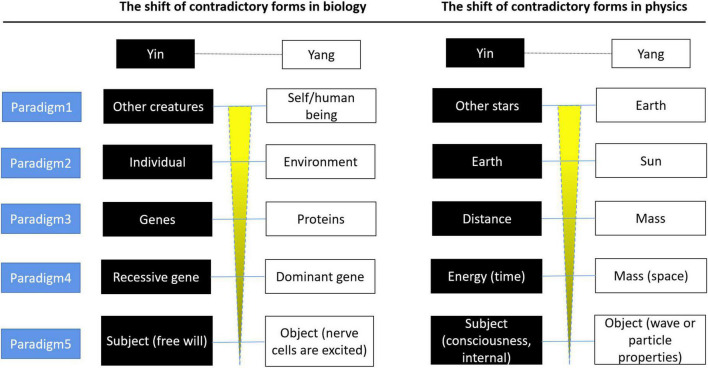
Contradiction forms of paradigm in different stages are from obvious to hidden, from intuitive to basic in terms of perception. Objects in completely different physical forms (For example, genes and the earth) can be reduced to two contradictory (Yin-Yang) perceptual states. Yin: shows characteristics in perception like abstract, internal, hidden, defensive, passive etc.; and Yang: On the other hand, shows characteristics in perception like concrete, external, prominent, aggressive, positive etc. On the contrary, in order of precedence, Yin is in front and Yang is behind (Yin-Yang instead of Yang-Yin). For example, the exterior of a tree comes from hidden roots. Genes are the determinants of heredity, not proteins. Therefore, the causal view of holistic philosophy is internal, and the material view seeks external cause and effect. **Paradigm l:** In the most intuitive state of perception, according to external physical form, human beings are the most advanced creatures in nature and are very special beings. Thus, the self/human has core status (exhibiting abstract features of “Yang”) relative to the other creatures, while other creatures are relatively insignificant (exhibiting abstract features of “Yin”); the other stars revolve around the earth and are therefore passive and active relationship (Yin and Yang, respectively). **Paradigm 2:** The theory of natural selection suggests that the individual needs to adapt to the environment, so the individual is in a passive status (Yin) and the environment is in an active status (Yang); the heliocentric theory proposes that the earth revolves around the sun, so the sun is active (Yang) and the earth is passive (Yin). **Paradigm 3:** Genes are the internal determinants of biological traits (Yin) with hidden characteristics, while external traits are mainly presented by proteins (Yang); two descriptors in the equation of universal gravitation, F = Gmlm^2^/r^2^. Mass (m) is perceived first and distance (r) second, so distance is Yin and mass is Yang (Just like a cup, the surrounding outer wall is focused by perception firstly, and the empty part inside is focused secondly, but the two together can hold water as a cup). **Paradigm 4:** Mendel proposed the most basic genetic law was based on dominant gene (A) and recessive gene (a). The recessive gene (Yin) was in a hidden status compared with the dominant gene (Yang); in the theory of relativity (E = mc^2^), the relationship between two fundamental descriptors, mass exhibits concrete characteristics (Yang), while energy is an abstract state (Yin). **Paradigm 5:** Matter exhibits remarkable and concrete characteristics (Yang), consciousness is abstract and hidden (Yin), you can’t see it but you can feel it. Both physics and biology reveal an inseparable relationship between matter and consciousness or subject and object. It should be emphasized that this empirical division of Yin and Yang according to experience and perception is not absolute. For example, in rare cases, proteins can also serve as genetic material, reflecting a mutually inclusive relationship between contradictions.

In other words, in the development of cognition, the contradictory form is always from the most intuitive and obvious form (in terms of perception) to the most subtle and hidden form of contradiction (this relates to the relationship between two basic descriptors of a theoretical paradigm. For example, natural selection explains evolution by describing the relationship between individuals and the environment, and the theory of relativity describes the relationship between mass and energy). This blurs the boundary between the subject and objects and is beyond the distinction of external physical forms (Table 1). Some experiments reveal inclusive relations between the two sides of the contradiction, and the contradictory form is ultimately the manifestation of the most basic relationship between the subject (consciousness) and the object (matter). This is from the antagonistic relationship established in the earliest view of the matter to the phenomenon that they contain one another, but a theoretical framework that can be proved scientifically describing the unified relationship between matter and consciousness (or object and subject) is still lacking (Figure 4). For example, our most conspicuous perception is that the self is a unique and conscious species of animal in the universe. The revealed reality or phenomenon of scientific activities always tends to take place in the opposite direction, which is a natural tendency for unifying the differences and unique characteristics in an external physical form that one feels naturally at the beginning. This trend and regular development process cannot be separated from the state of perception as reference frames, and they move in a formalized non-linear way. From this point of view, scientific cognition itself (the reality or phenomenon revealed) based on perception can be deduced in a paradigmatic way.

**TABLE 1:** The paradigms in different stages of scientific cognition show a regular tendency in terms of perception.

	Core theories listed represent phenomena or reality revealed by different stages of paradigm		The shared meaning abstracted from the revealed phenomenon or reality (in terms of perception rather than specific mathematical or logical descriptors)	Diagrams used to represent the state of perception abstracted from the phenomena and its evolution tendency
	Biology	Physics		
Paradigm 1	Humans are unique in nature	Geocentrism; The earth is the center of the universe	Depend on the distinction of external physical forms, the relationship between subject and object is antagonistic. It was believed that the self/human has a special status or location in the universe. The spheres of different shapes and colors represent the difference in physical form (mass, volume, speed, anything that can be described) between the self/human and external objects. The big yellow circle represents subject, and the surrounding spheres are around it, indicating that self/human has a strong feeling that self/human is very special in universe.	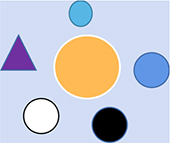
Paradigm 2	Theory of natural selection (individuals need to adapt to environment)	Heliocentric theory	Human beings have no special or core status in the universe and are common species in nature. The heliocentric theory suggests that the earth revolves around the sun. The circle in the center of the diagram became significantly smaller and lighter, indicating that the feeling that human is very special is weakened compared with the former paradigm.	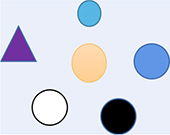
Paradigm 3	Ligand and receptor interaction. Laws of inheritance based on dominant and recessive genes	The theory of gravity	A linear cognitive model is established based on the principle of interaction and determinism is gradually formed. Physics takes force as the basic concept; while biology forms information transmission based on the interaction between ligand and receptor. Make a mathematically descriptive connections between descriptive quantities/characteristics, but the relationship between time (energy) and space (mass) is antagonistic.	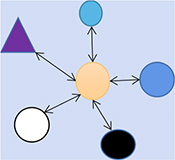
Paradigm 4	A universal network of interactions between molecules	The theory of relativity	This paradigm further expands the scope of universal connection, time and space are inseparable and not antagonistic relation. On the physical level, there was a universal connection between objects, and the abstract concept of “field or network” was more suitable to describe the real connection pattern between objects. Meanwhile, determinism reached its peak, but subject and object are opposites. The use of grids to describe the interaction between objects in the diagram is used to represent ubiquity and abstraction.	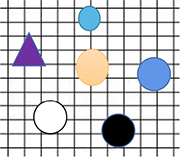
Paradigm 5	Mirror neurons were found	Wave-particle duality and quantum entanglement	At the micro level, the cognition of the objective world is challenged; the scope of connection is further expanded, not only is there a universal connection between the physical level, the contradictory nature (wave or particle characteristics) of objects is inseparable from the subject. We are more confused about the nature of consciousness than ever before. For example, in mirror neuron experiments, relying solely on brainwaves cannot distinguish between object and subject, showing inclusion relation of object and subject beyond external physical form. In the diagram, the observer is inseparable from the state of objects being observed. The different features of exterior objects are represented by contradictory nature (black or white). A grid describes an indivisible abstraction connection between subject and object, external and internal. But a scientific theoretical framework to describe the relationship between inner and outer is still lacking.	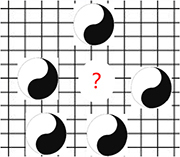

We think it contains the following three features: (1) In terms of perception, the contradictory form of different paradigm (for example, the theory of relativity describes the relationship between mass and energy) goes from obvious to basic, from kinds of distinction on physical forms to subject and object (external and internal) inseparable. (2) The development of scientific cognition is also a synchronous process of breaking the feeling state that the self/human is very special in terms of external physical form distinctions. (3) The trend of reality paradigm shift dependent on the state of perception, in other words, state of consciousness acts as an abstract frame of reference to determine the occurrence of reality, reality is not a purely objective process observed by the observer. There are no objective laws independent of perception.

Second, referring to external physical form, different disciplines and theories are independent. However, there are obvious similarities in the abstract meanings of phenomena revealed by paradigms in different disciplines. For example, the concept of mirror neurons in biology shares similar abstract implications to the phenomenon of quantum wave-function collapse in physics. They both revealed the inclusive relations and inseparable relationship between two aspects in a contradiction (subject and object).

For a long time, science has been defined as an objective description of the laws of nature. We are only objective observers exploring objective laws. Biology and physics, based on reductionism, seek to explain the universe through the motions of microscopic cells and quanta, but why do scientific theories (the reality or phenomenon revealed) show regular paradigm shifts and significant similarities in perception between different disciplines (Figure 4)? We argue that this implies that scientific cognition itself may not just be an objective and independent process of observation but a synchronous process that constantly breaks down self-particularity (based on external physical forms) perception states contained in a more unified framework of laws, reflecting the natural trend for *everything* to move from opposition to unity. Hegel did not separate nature from history. For the first time, he described the natural, historical, and spiritual world as a unified process and tried to reveal the regularity and objectivity of its movement and believed it was the contradiction that led to the change and development of movement (Hegel, 1994, 2004). This view is also supported by our discussion of trends in scientific cognition in this section, as follows: the laws of nature and the laws of society are an inseparable process, which can be seen as the paradigm shift process, the reality or phenomena revealed by scientific theories is inseparable from perception state, and the occurrence of reality is not just governed by an external objective law.

It is worth noting that the dominant view of our current “human-special” concept is that only humans or higher animals have consciousness. Thus, the distinction between the inanimate and the living, matter and consciousness, and external and internal may also be a problem we need to address.

#### The next possible paradigm: the unity relations of matter and consciousness at the perception level

To reconcile the gap between body and mind, Solms (1997) argues that the solution to this problem must reduce its psychological and physiological to a single physical abstraction (Solms, 1997, 2014). From the perspective of holistic philosophy, the development trend of scientific cognition also supports this view. Depending on perception, scientific cognitive theory itself presents a regular paradigm shift trend. Based on the discussion in the previous chapter, we try to deduce the next possible paradigm or framework and propose scientific hypotheses that can be verified through experiments.

The external (object) and the internal (subject) are indivisible (showing that both sides of the contradiction are inclusive). For example, the wave and particle properties exhibited by a quantum are inseparable from the observer, and the observing subject and object in the mirror neuron cannot be distinguished by external forms. The paradigmatic shift trend of reality is from self/human-centered cognition to the conclusion that the self is not special, from the external physical characteristics of antagonistic relationships to unity (inseparable from perception), and it shows a tendency to distinguish objects beyond external physical forms. A paradigm that can be mathematically described must be represented in contradictory forms (e.g., mass and energy, dominant and recessive genes), and the contradictory forms that construct the new theory increasingly tend to be the most basic and the most subtle forms distinguished by perception.

Based on the above three features, we propose the next possible cognitive paradigm: a more abstract presentation form of contradiction that transcends physical form, which is distinguished by perception to describe the shift of reality in a paradigmatic manner. This possible next paradigm is the basis of holistic philosophy, marked by the Taiji Diagram (depending on conscious experience, all existence is summarized as contradictory perception states of Yin-Yang, replacing concrete physical features). The two sides of contradiction are interdependent, interlaced, and inter-transformed. This interpretation is based on our understanding of the *Tao Te Ching*, and its rationality needs to be supported scientifically (Table 2).

**TABLE 2:** Describing the evolution of reality in terms of perceived abstract contradictions (Yin-Yang) may be the next paradigm reconciling the antagonistic relationship between matter and consciousness.

	Core theories listed represent phenomena or reality revealed Y different stages of paradigm		The shared meaning abstracted from the revealed phenomenon or reality (in terms of perception rather than specific mathematical or logical descriptors)	Diagrams used to represent the state of perception abstracted from the phenomena and its shift tendency
	Biology	Physics		
The supposed paradigm 6	What is the function of subjective experience? The free will is at odds with causality based on material view.	How consciousness causes wave function collapse (affecting the occurrence of reality/order)	Matter and consciousness are not opposites but are unified in perception, they are two different states of perception (it is represented by the abstract Yin-Yang). Both the subject and the object are represented by the abstract Taiji diagram, and there is no external physical form distinction between subject and objects (self/human is not special in terms of external physical form). The dotted box in the diagram indicates that the essential state of things is relative and inseparable from perception. Describe fate or reality in terms of perception (experience) is the subject of this paradigm. Fate or reality can be described in a paradigm shift way and inseparable from perception (irrational side)? This paradigm also happens to be the cognitive basis of holistic philosophy.	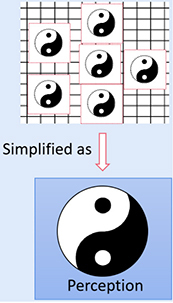

We propose that matter and consciousness are not two opposite existences but two completely different contradictory states of perception. They can be represented by the abstract Yin-Yang (Yin: has characteristics in perception like abstract, internal, hidden, defensive, and passive; Yang: has characteristics in perception like concrete, external, prominent, aggressive, and active). They can be distinguished and are unified at the perception level. Since they are two sides of the same coin, this can be demonstrated by the influence of “internal” state of consciousness on the “external” occurrence of reality.

**FIGURE 5:**
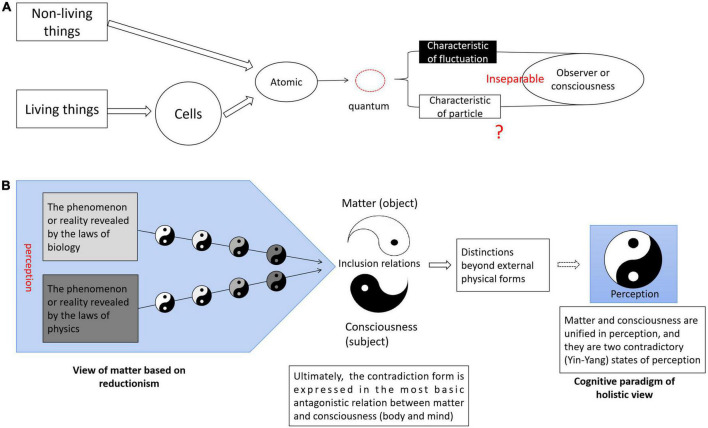
Dilemmas of consciousness in biology and physics may be reconciled by the holistic cognitive paradigm. **(A)** Reductionism seeks to explain everything (the theory of everything has nothing to do with perception) by understanding the motion of microscopic atoms (quanta). Phenomena revealed by quantum mechanics, such as wave-particle duality, challenge this basic cognitive paradigm. The nature of a quantum (wave or particle) is inseparable from the observer (or consciousness). However, further mathematical descriptive theoretical framework based on this paradigm has not been established. **(B)** The reality revealed by biology and physics presents a regular paradigm shift trend and has similarities in terms of perception. The contradiction form (paradigm) in terms of perception gradually changes from obvious to the most basic, from concrete to abstract. Both disciplines ultimately reveal, respectively, the limitations of cognitive paradigms based on material view, reflecting the inclusive relationship between matter and consciousness (It was first believed that the objective material world had nothing to do with perception, and the two were opposites, using different ways of describing human society and nature). Following the previous paradigm, we conjecture that the next paradigm will not be described in concrete physical form, but in the paradigm evolution of reality based on perception and experience. In this paradigm, individuals are defined in terms of different fates and realistic outcomes rather than physical forms.

Modern biology and physics are based on material views and reductionism, whereby scientists seek to explain the universe through an understanding of the laws of microscopic cells and material “entities” such as quanta. However, the most fundamental assumptions of this cognitive paradigm have been challenged by some of the phenomena and most fundamental problems revealed by recent science (Figure 5A). For example, biology is confused about issues like free will and causality based on space-time, the phenomenon of hyperspace-time quantum entanglement discovered by physicists, and how consciousness causes wave function collapse (affecting reality). These phenomena reveal the inclusive relations between two sides of contradictions (subject and object, consciousness and matter, internal and external), but the descriptive theoretical framework for reconciling (unifying) the opposite relationship of matter and consciousness has not been established. We propose that the cognitive paradigm of a holistic view based on perception provides the possibility to reconcile these paradoxes. From this point of view, the confusion of biology and physics about consciousness can be unified into one same problem: how reality is created depends on the state of perception (not subjective intent and purpose; Figure 5B). We will elaborate on this hypothesis in the following sections.

### The paradigm-shifting trends of reality depend on irrational feelings

#### The occurrence of reality is inseparable from irrational feelings

Panksepp’s work led to the recognition of the importance of emotion in the study of consciousness and he coined the phrase Affective Neuroscience in 1991 (Panksepp, 1992; Davis and Montag, 2018). To distinguish it from rationality more obviously, we used the word irrationality rather than sensibility (in this manuscript these two words have the same meaning) to refer to the sense of reasonableness as a result of experience, the experience involving emotion with no thinking. According to Solms, if we want to coordinate psychological and physiological aspects to explain consciousness, we must focus on the feeling and experience.

If the internal experience of having a memory and the neuronal assemblage embodying that same memory (pictured externally, through optogenetics, for example) are two realizations of a single underlying thing, then what is “memory” itself made of? The answer is that it is abstracted from both manifestations. Memory is not a stuff; it is a function. If we want to identify a mechanism that explains the phenomena of consciousness (in both its psychological and physiological aspects) we must focus on the function of feeling, the technical term for which is “affect.” That is why it is easy to agree that consciousness is not just another cognitive function (Solms, 2018).

In the first paragraph of this section, we mentioned Thomas Kuhn’s suggestion that scientific cognitive processes are paradigm-shifting processes that cannot be logically inferred. We believe that the shifting of scientific paradigms depends on the irrational side of feelings. It is an irrational tendency of movement, irreversible and relative, and is also an objective movement form that exists in contrast to logical/rational characteristics. For example, in the ancient days of human civilization, people advocated for “an eye for an eye, a tooth for a tooth,” which they deemed reasonable. Now, if someone hurts another person with an axe, instead of punishing them in the same manner, we imprison them. This reality evolves depending on the abstract and irrational “sense of reasonableness.” From simple linear causal to non-linear compensation, the development trend of reality is formed. This trend cannot be independent of perception and experience, as there is no logical or physical connection between imprisonment and the axe. It cannot be strictly quantified, but it shows a clear trend at the perceptual level. If we turn this phenomenon upside down, we will find that it is extremely disharmonious at the feeling level and will cause chaos and collapse in reality, which does not conform to the developmental trend of things, and thus the resistance encountered will grow.

Cognitive trends abstracted from phenomena revealed by different paradigms share similar characteristics. New paradigms can be more consistent with the coordination of irrational feelings than previous ones. These trends make sense at the level of perception (a relative concept that can only be defined concerning previous paradigms) but not the other way around. At the level of physical forms, some are even as far apart as an axe and imprisonment. For example, waves and particles in physics are completely unrelated in a way similar to stem cells and differentiated cells in biology, and yet, they are unified/similar in irrational feelings and represent a potential state (which can become any specific state) and a specific state of beings (abstract and concrete; Yin and Yang, respectively, not the other way around). The irrational feelings influence the occurrence of an external reality. According to holistic philosophy, the description of the world depends on perception rather than observation, which means that the self and *everything* are connected and indivisible from perception (the distinction between subject and object is independent of physical forms). Therefore, the irrational feelings we experience are not subjective or individually owned, but one of the objective tools for creating reality.

#### The reference frames for the occurrence of reality are perceptual states rather than external physical entities

In the process of scientific exploration, we are used to the material view of cognition that sees the self/human as an observer, and we separate social activity from scientific exploration. Whereas human social activities focus on inner feelings and experience, scientific activities are considered to explore objective laws of nature as observers. However, we find that scientific laws that are regarded as objectives show a regular paradigm shift trend that cannot be separated from irrational perception, and finally find that subject and object (or consciousness and matter) cannot be separated. This shows that there is no external objective law or material world separating “inner” perception, and the absolute assumption of the self as a pure observer is also limited. There is a more holistic framework that can unify the antagonistic relationship between consciousness and matter (subject and object), and we propose that holistic philosophy offers such a possibility.

Empiricists believe that human knowledge of the world comes from human experiences, while rationalists believe that human knowledge comes from human reasons. Kant, on the other hand, reconciled the two views to some extent. Kant believed that knowledge is obtained by human beings through sense and reason at the same time. Experience is necessary for the generation of knowledge, but it is not the only factor. Rationality is needed to convert experience into knowledge. We further extend this definition in this study (Kant, 1949).

Let us illustrate this abstract meaning with an example. If we watch a basketball game, we might think that spectators have no impact on the results of the game. In fact, from a holistic perspective, the preferences of perception (irrational “sense of reasonableness”) can influence the paradigm/style of the game in discontinuous ways (reality occurrence in paradigm-shift ways). For example, a National Basketball Association (NBA) offense might first shift from advocating a near-the-basket offense to a mid-range jump shot and finally shift to a focus on shooting from outside the three-point line (the evolution of reality was initially the most obvious pattern of rationality, the closer the players were to the basket, the easier it was to shoot, based on observation or rationality). This process is the change in which the spectator can empathize through feeling with the player (individual), feel/seek the path of least resistance to attack, and not just as an objective spectator that has nothing to do with the game, the reality tends to the path formed by the game of two contradictory “forces or tools” (the state of feeling as a spectator or as a player). Without empathy (relying on perception rather than observation), the spectator will not be able to understand or predict the paradigm shift trend of this game (reality), and reality will always be subject to this contradictory state and evolve regularly and periodically. This process is a gradual movement from opposition in rationality to unity in irrationality, from the outside to the inside, from the most intuitive form in perception to a covert one (the dominant offense evolved from near the basket to the three-point line, just as the cognitive paradigm of science is gradually shifting from the outside world to the inner world), which describes a holistic framework for the evolution of the reality. This is the basic descriptive feature of holistic philosophy, which relies on irrational perception and is abstract, rather different from reductionism.

Some successful old-school coaches may not understand the development trend of this phenomenon and still yearn for the game’s original shoot-under-the-basket style. This is because they are used to the perception of preferences as spectators (the spectator always tends to like an intense game) while ignoring the feeling of the optimal offensive choice as a player in the game (lack of empathy based on perception). As a result, they have no way of predicting where the game paradigm is going. Just as the scientific system based on reductionism *ignores the objectivity of perception* and regards the self/human as an independent observer (or treats experiences and feelings as subjective beings, independent of the physical world), it cannot continue to rely on the basic assumptions of observation and rationality (the view of matter as opposed to consciousness) to explain the origin of macroscopic order. This cognitive paradigm develops so habitually that we take it for granted, until it is challenged by paradox phenomena revealed by biology and physics. Therefore, we think that the evolution of reality paradigms is always driven by these two abstract contradictory tools or two contradictory statuses of feeling.

It must be emphasized that this phenomenon does not apply only to the activities of human social activity but to the scope of the evolution of *everything* (the object described by the holistic view is reality itself). These two tools (Yin and Yang, rational and irrational perception) are two objective tools for creating reality, and not the subjective form that belongs to the individual. In a holistic view, the individual is regarded as non-special, and the subject and objects are connected, indivisible, and unified through perception. Self/human are also a contradictory existence connected to perception, so they cannot be independent of natural laws as observers, and their perception state as the reference system participates in the occurrence of reality.

## The objectivity of perception

### Contradictory nature that can be experienced by perception is the objective form of being

In this chapter, we will discuss the theoretical basis on which reality can be described objectively by perception. The split-brain experiments shed some light on the contradictory nature of consciousness. In the 1940s, scientists cut the corpus callosum of epilepsy patients who did not respond to medical treatment. However, split-brain individuals whose corpus callosum has been incised have a distinct feeling of division or the act of division. For example, when Sperry injected a command to raise one’s hand or bend one’s knee into the left side of the split-brain, the patient’s right side obeys the command but the left side does not; there are many other similar contradictory behaviors (Gazzaniga, 2005; Volz and Gazzaniga, 2017; de Haan et al., 2020).

According to Hegel, everything contains a contradiction, which is the root of all movements. At the same time, he also regarded the development of contradiction as a process from in-itself to self-action. Opposition, distinction, and unity are different stages of contradiction development. Only after experiencing the contradiction of opposites will the unity of new contradictions be realized; opposite contains unity, and unity also contains the opposite. This dialectical thought is similar to the connotation of the Taiji diagram (Hegel, 1983, 2004).

Intuitively, though, internal perception is seen as subjective, it also has an objective nature of contradiction beyond the control of any individual. It does not belong to anyone/existence, but it is dynamically connected with any existence on the level of perception. Therefore, the contradictory nature of being experienced by perception is actually the source of creation (it divides existence into two contradictory perceptual states, Yin-Yang, which are connected with perception), which exists dynamically in an abstract but in a perceivable way and is not a subordinate feature that we can induce from material world phenomena, and this subjective level of objectivity is one of the bases for the holistic philosophy to describe the development of the evolution of reality.

Perception, which is perceived as subjective and is considered unique to human beings or higher animals, is remarkably objective. For example, if told not to think about what a tall, red spruce looks like, a person cannot help but imagine it. If we flip a coin 10 times and every time it comes out heads, we will become increasingly curious and try to find the cause and effect, and if the results become increasingly balanced, we will experience the same kind of confusion. Once science finds and defines a rule, for example, that a random mutation of a base is selected by nature to lead to the evolution of a species, it also means that this statement will no longer be objective. We will continue to master targeted base-editing techniques to modify living species and reality will always move in the opposite direction, since objective observation and feeling preference cannot coexist. As science advances, we acquire even more sophisticated and high-resolution photography, but by contrast, forms of artistic expression begin to seek more abstract ways of expressing the uniqueness and meaning of their existence, we worship the spotlight of the stage, but once it reaches its peak, the noise makes feelings pursue being ordinary and vice versa. This perceptual contradiction is the most basic objective feature of the inner. The holism cognitive paradigm reduces everything to an abstract state of Yin and Yang, which is not merely the nature of external self-expression, but inseparable from internal perception or conscious experience. Therefore, Yin-Yang is regarded as the co-creation of subject and object and is inseparable from perception. On the other hand, the material view focuses on describing the objectivity of external physical properties (describing the object from the relationship of opposites), ignoring the basic unity relationship between internal perception and external.

This state of contradiction is a fundamental pattern beyond the control of any individual and, at this level, everything is connected in perception rather than demonstrating an external space split, and this suggests that, contrary to our intuition, we are not masters of perception; a new self-model based on the principle of Taiji expounded on the contradictory nature of the self (Wang et al., 2019; Wang and Wang, 2020).

Of note, when we interpret the relationship between matter and consciousness, we define consciousness as one of the abstract states of perception, as opposed to the states of concrete perception that form. For the most part, we use the word perception alone as the creator of all things, similar to the concept of Tao, but this description is not appropriate. The creator cannot be described by language or any concrete measure. If it is defined as one of these, then it means that it is no longer the other, and it loses its totipotency. We still use this word, in part to highlight the indivisibility of creation and perception, but habit is what keeps us from finding more appropriate words to replace this word. In other words, perception is not owned by the individual. What defines us as individuals are the different realities (active or passive, goals achieved or not achieved, the different outcomes of events experienced by individuals) that we perceive. Since they can only be experienced and not observed, we intuitively think of them as belonging to the individual/human, not to other animals or non-living things, forming a worldview of matter as opposed to consciousness. The view of matter defines the individual in terms of biological or physical characteristics, while what determines reality is the contradictory nature of inner perception, which is beyond the control of the individual.

We believe that this interdependence of internal and external at the perception level is the most fundamental form of movement. At this level, everything is naturally connected and in perpetual motion, resulting in reality never occurring in a static, linear, or causal manner, detached from the perception of the subject. At any time, in the form of potential indivisible contradictory non-linear fluctuations, the dynamics alternating between the law and the irregular are the basic characteristics of the universe, nature, society, and other evolution of reality.

### Explanation of quantum entanglement based on holistic philosophy

Our explanation for why the hyperspace–time entanglement occurs between two particles born in the same system is as follows. (1)We will ignore the objectivity of the inner and simply seek the connection between “two particles,” from the meterial view. It is not that two-particle entities are mysteriously interacting in hyperspace. As two ends of a contradiction, internal and external are born at the same time and are inseparable. The quantum state (topspin or backspin) observed by the observer is inseparable from perception, so they are not constrained by time and space. From a holistic view, the “particle” of a material entity independent from perception neither exists nor can be described, and beings can only exist in terms of contradictory physical properties (up and down, right and left, black and white, etc.) that cannot be separated from perception. Different observers are essentially different states of a conscious experience.

(2) In the previous section, we argued that the contradictory nature of inner perception is the most objective form of existence and that the material entity, which although gives us a very real objective sense, is not the most essential existence, objectivity is relative. It is only the side that is conspicuously perceptible to perception (Yang: a conspicuous, specific, prominent perceptual state). The occurrence of quantum entanglement depends on the rational “sense of reasonableness” (it is reasonable for external phenomena to remain symmetrical if they are based on observation or rationality).

(3) In other words, if we regard that the symmetric descriptors topspin and backspin are born at the same time as an occurrence of the result of an event (reality), the reference frame for quantum entanglement occurrence is the rational “sense of reasonableness” of perception. What we see is only the conspicuous aspect of conscious perception (Yang). But according to the material view, we only mistakenly regard it as an observed process rather than a happening process that is inseparable from perception.

(4) The other irrational side (a state of conscious feeling that depends on the experience of the observer) is hidden and dynamic and is difficult to describe using explicit linear equations (Yin: a hidden, abstract state of perception). For example, just as one plus one equals two is generally accepted from rationality and logic, we find that in social activities and specific situations, one plus one can have any possible outcome in different realistic situations. However, this conscious experience is dynamic and needs to be in a relative situation to form a generally recognized (objective) state of reasonable feeling. It is in a hidden and unstable status that cannot be absolutized. However, that does not mean that it is not one objective side creating the order of reality.

**FIGURE 6:**
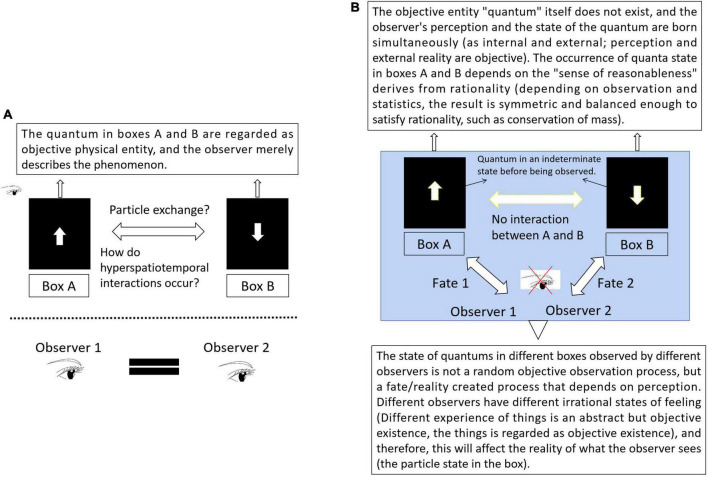
Interpretation of quantum entanglement based on holistic view. **(A)** Material view. Based on the difference in external physical form, observer and objects are opposites. External phenomena are irrelevant to the observer (or perception/consciousness). It cannot explain the hypertemporal effects of quantum entanglement. **(B)** Holistic view. Depending on perception, observer and object are unified. Perception is co-created by subject and object. In other words, what we intuitively regard as subjective, individual and abstract being (experience and feeling) can be described scientifically (Yin-Yang) and used as frame of reference to describe the occurrence of fate or reality of the observers, from the material view, the state of the quantum in boxes A and B is an objective random process.

Therefore, if the above explanation is correct, what we need to demonstrate is that the quantum state observed in different places is not random (the reality of the observer; which is regarded as random according to material views). It can be affected by the state of perception of the observer and, more specifically, the state of conscious feeling formed by experience on the irrational side (Figure 6). Shaping the specific state of perception (irrational side) of the observer can affect the reality of the observer (the observed probability of upspin or downspin).

### A preliminary discussion of hypothesis proof

From a material view, the connection between objects requires force as a medium, and the force originates in the interchange of microscopic particles. Therefore, we are puzzled by the spatio-temporal nature of quantum entanglement. In a holistic view, the nature of connections between objects depends on irrational perception (for example, the fact that trees and grass, as well as the sun and moon, can produce similar Yang and Yin states of perception, rather than the other way around, is a fundamental property of perception, despite their differences in physical level). Unity in perception means that connection does not need any material medium. Taking the double slit interference experiment as an example, the particle’s choice to take slit A or slit B as the path is regarded as random under a material view. Researchers shaping the specific state of consciousness of the observer (irrational side, depending on individual experience) will not influence the result of the observer. Otherwise, doing so would affect the result without any material medium participation. Due to space limitations, we do not discuss further details of the experimental design in this study.

Many scientists believe that consciousness is the key to the collapse of the wave function, but we still need a model that can be scientifically validated. According to our previous analyses, existence is manifest in a contradictory way that is inseparable from perception. It is a comprehensive feeling state of the connection between objects generated based on experience and a dynamic superposition of irrationality and rationality (for the sake of the statement and later experimental verification, we replace Yin-Yang with rationality–irrationality), rather than objective material entities.

What we want to emphasize is that in the perception of the relationships between objects, which is also the essential/objective state of connection between objects in a holistic view, the abstract state of perception is a state of superposition of rationality and irrationality (the mutual inclusion relationship in Taiji diagram), and the rational conclusion is only a one-sided illusion, a state in the conspicuous feeling state (Yang). Just as feelings of self tend to focus on physical features, body shape, and needs, rather than on the other side, empathy (or we can say both selfishness and selflessness) is the natural contradiction of the self, but selflessness is often in the hidden status of paradoxical side (Yin) as it forms a scientific cognitive system based on rationality/physical forms. However, the development trend of reality/paradigms leans toward the direction pointed out by this abstract irrational perception side, as we discussed in the previous chapter, on the development of scientific cognition. Thus, by shaping and influencing the irrational side of the observer’s state of perception, the reality of the observer can be described and predicted by the researcher. This process is considered random or indescribable based on a material view.

## Conclusion

Physics and biology together reveal some puzzling phenomena related to consciousness. Although they are manifested in different forms, they both imply the interaction and indivisible features between matter and consciousness, which cannot be reconciled by the cognitive paradigm of materialism and reductionism which describes matter and consciousness in opposition. In this study, we propose that a perception-based holistic view cognitive paradigm is a potential solution to reconcile this dilemma.

The material view explains phenomena based on forces, and the connection between objects depends on the exchange of microscopic particles. We can explain some natural phenomena from the level of physics and form causality based on time and space. Therefore, the definition of causality in the material view requires a physical medium and is confined to space and time, which we take for granted. However, this cognitive paradigm fails to explain some consciousness-related phenomena such as, how does the mind lead to physical changes in the body, how does consciousness lead to the collapse of wave functions, and why does quantum entanglement appear to be independent of space and time? In these phenomena and effects, we cannot observe any medium involved, so this violates the causality based on the material view, leading to a paradox or gap in interpretation.

On the contrary, the holistic view relies on the conscious experience to distinguish the thing into a Yin–Yang perceptual state, subject and object can not independently described at the perceptual level. Yin and Yang, in other words, do not simply refer to the characteristics of the object itself. It is inseparable from the subject’s descriptive means (different conscious experiences), but intuitively, we think of conscious experience as subjective and belonging to the individual. The cognitive paradigm of holism does not describe matter and consciousness in opposition, or ignore either of them, but regards them as two objective states of being that can be distinguished by perception (e.g., concrete or abstract; rational or irrational), unified in perception. This has two meanings: (1) The inner is as objective as the outer, (2) because they are two sides of the same coin unified in perception, the reality (outer) and the inner can interact, which can be seen as a state switch manifested by the objective nature of consciousness. To be more precise, the occurrence of an event (external) from with the sense of reasonableness that come from irrationality as a reference frame, which does not require any physical medium and is not constrained by space and time.

This leads to a significant difference. The material view opposes the subject (internal) to the object, which is described as an objective physical being that is same to all observers. The description of the holistic view describes the subject (conscious experience) and the object (external) together on the basis of perception. Therefore, it describes the results of events rather than physical features, such as the reality of an observer. The two cannot be independent or described in opposition because the reality varies with the observer’s state of consciousness (Table 3).

**TABLE 3:** A contrast between the material view and the holistic view cognitive paradigm.

	Material view	Holistic view	Additional remarks
Description method	Rely on observation, rationality (it can be referred to by an asterisk 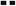 ).	Rely on conscious experience, irrationality. (it can be referred to by an asterisk 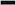 ).	The materialistic view is the cognitive paradigm to which we are most accustomed, and the underlying assumption of human beings as observers is not even questionable, while the holistic view, which relies on perception, is easily ignored or regarded as the ownership of individuals.
The relationship between matter and consciousness	Matter and consciousness (or object and subject) are opposites.	As two objective states of being that can be distinguished by perception, unified in perception.	In terms of the objectivity of perception, the abstract perception of Yin-Yang is objective existence, just as the objective features of external objects is observed, but it is an abstract form.
The object being described	It describes external physical characteristics, and has nothing to do with perception.	It contains both subject and object; describes the happen probability of an event (reality, object) of an observer (subject).	In the holistic view, the internal state of conscious perception affects reality, so a precise description must include subject and object.
Different emphasis on objectivity	Focus on the objectivity of external physical form. Mass, size, momentum, etc.,	Focus on the objectivities of perception. (1) Contradictory nature; (2) Irrational conscious perception; (3) Always seek a more harmonious state of being (a relative concept that depends on perception).	Objectivity is the basis on which a cognitive paradigm is constructed. We argue that objectivity has two different way of description, focusing on the external and the internal respectively, satisfying rational and irrational “sense of reasonableness”.
The contact between objects	Particles exchange (physical medium that can be observed) can form different forces, which mediates the connection between objects.	Depending on conscious experience (it’s an abstract being that can be experienced but cannot be observed), the things are summed up as Yin- Yang perceptual state, which is not arbitrary and can form a common irrational feeling.	In the interpretation of the origin of all things (the habitual description of the material view) or the evolution of reality (the habitual description of the holistic view); they try to explain through microscopic quantum and abstract Yin- Yang respectively, inseparable from conscious experience.

For example, in the experiment of quantum entanglement, from the material view, the observer only describes the state of the particle objectively, which has nothing to do with the conscious experience of the observer. Therefore, the state of the particle observed by the observer is considered to be random. According to holism, the observer’s irrational conscious state will affect the probability of the observed particle state, and the conscious state is closely related to life experience. In other words, the perceived asymmetry of what is regarded as an objective being is the root cause of order (reality does not happen randomly), and this makes it possible to scientifically prove the cognitive paradigm of holistic view philosophy. Due to space constraints, we do not discuss the details of the experimental design here. We elaborate it in detail in another article published in the preprint (doi: 10.31219/osf.io/c3neq).

Objectivity is the foundation on which a discipline or theory is built. The holistic cognitive paradigm is based on the objectivity of perception. Although it is abstract, it can be described scientifically (do not rely on reason and logic). According to our understanding of the *Tao Te Ching*, in this study, we introduce three basic objective properties of perception. (1) Contradictory nature. Conscious experience, though intuitively viewed as subjective, belongs to the individual. On the contrary, perception presents a contradictory and objective nature beyond individual control. What the individual experiences is only the sensory state (Yin or Yang; positive or negative) formed by the different reality determined by this objective nature. This means that the observer’s reality is descriptive, not random and unpredictable, but indescribable from the viewpoint of force and reductionist. Let’s use the analogy of the relationship between DNA and living things. On the surface, we believe that organisms possess DNA, but in fact, this relationship is inverted. DNA determines biological traits according to objective genetic laws, and individuals only show different biological traits that have been determined.

(2) Irrational conscious experience. The irrational conscious experience can summarize things into Yin–Yang states of perception. This induction is not arbitrary but has an objective standard, which is not essentially different from the description of external physical objectivity that we rely on reductionism, except that one is abstract and the other is concrete. (3) Always seek a more harmonious state of being (a relative concept that depends on perception). It determines that the development of reality has a relative direction and trend, for example, scientific cognition itself shows a regular inertial development trend.

The holistic cognitive paradigm also provides the possibility to coordinate the contradiction between determinism and non-determinism. Due to the objectivity of perception, the occurrence of reality is descriptive and regular. However, this does not mean that the observer’s reality is completely determined (determinism), it is probabilistic and independent of perception. Since it is inseparable from perception, which always pursues a more harmonious state of being (the third objectivity), reality will change due to the switch of the observer’s state of consciousness, reflecting the subjective initiative of consciousness.

For example, according to holism, we can describe a person’s reality in terms of conscious experience. If we just observe (in fact, this assumption is not entirely accurate because observation and feeling contain each other and can only be said to have a minimal probability of influence), then this probabilistic description can be verified. However, if we tell the observer what is going to happen to him, the state of consciousness of the person who is told will change because perception is always pursuing a more harmonious state, and then the probability of his reality will naturally change. Due to the contradictory nature of consciousness, it refuses to be completely determined and it also rejects absolute disorder. For example, if we are told not to think about a big mangrove, we cannot help but imagine it. We think that there is no right or wrong distinction between the material view and the holistic view. The construction of these two cognitive paradigms originates from two different ways of looking at things (observation or perception) and forms two different sense states of rationality. The different cognitive paradigms and ways of solving problems (it is also a reality-creation process) have limitations and should be complementary (Capra, 2000).

In conclusion, based on the understanding of *Tao Te Ching*, a representative work of holistic philosophy, we (1) deduced the next possible cognitive paradigm from a holistic view through trends of scientific cognitive development and proposed a preliminary scientific hypothesis; (2) summarized the confusion around consciousness in biology and physics as the same problem (of how to describe the evolution of reality depending on perception) and highlighted that holistic philosophy can solve this problem; and (3) we provided a new interpretation of quantum entanglement according to holistic philosophy, which is falsifiable. As interdisciplinary propositions, different disciplines are trying to describe consciousness from different perspectives (Friston and Stephan, 2007; Arsiwalla and Verschure, 2018; Marchetti, 2018). We believe that combinations of approaches from these different disciplines in the future will help us uncover the puzzles related to consciousness.

## Data availability statement

The original contributions presented in the study are included in the article/supplementary material, further inquiries can be directed to the corresponding author/s.

## Author contributions

JC constructed the theory and wrote the manuscript. LC revised and edited the manuscript. Both authors approved the submitted version.
